# Metabolomics Analysis of Mesenchymal Stem Cells

**DOI:** 10.22088/IJMCM.BUMS.8.2.30

**Published:** 2019-06-20

**Authors:** Parisa Goodarzi, Sepideh Alavi- Moghadam, Moloud Payab, Bagher Larijani, Fakher Rahim, Kambiz Gilany, Nikoo Bana, Akram Tayanloo- Beik, Najmeh Foroughi Heravani, Mahdieh Hadavandkhani, Babak Arjmand

**Affiliations:** 1 *Brain and Spinal Cord Injury Research Center, Neuroscience Institute, Tehran University of Medical Sciences, Tehran, Iran* *.*; 2 *Cell Therapy and Regenerative Medicine Research Center, Endocrinology and Metabolism Molecular-Cellular Sciences Institute, Tehran University of Medical Sciences, Tehran, Iran* *.*; 3 *Obesity and Eating Habits Research Center, Endocrinology and Metabolism Molecular-Cellular Sciences Institute, Tehran University of Medical Sciences, Tehran, Iran.*; 4 *Endocrinology and Metabolism Research Center, Endocrinology and Metabolism Clinical Sciences Institute, Tehran University of Medical sciences, Tehran, Iran.*; 5 *Health Research Institute, Thalassemia and Hemoglobinopathies Research Center, Ahvaz Jundishapur University of Medical Sciences, Ahvaz, Iran.*; 6 *Integrative Oncology Department, Breast Cancer Research Center, Motamed Cancer Institute, ACECR, Tehran, Iran* *.*; 7 *Department of Biomedical Sciences, University of Antwerp, Belgium.*; 8 *Metabolomics and Genomics Research Center, Endocrinology and Metabolism Molecular- Cellular Sciences Institute, Tehran University of Medical Sciences, Tehran, Iran.*

**Keywords:** Mesenchymal stem cells, metabolic pathways, metabolomics, systems biology

## Abstract

Various mesenchymal stem cells as easily accessible and multipotent cells can share different essential signaling pathways related to their stemness ability. Understanding the mechanism of stemness ability can be useful for controlling the stem cells for regenerative medicine targets. In this context, OMICs studies can analyze the mechanism of different stem cell properties or stemness ability via a broad range of current high-throughput techniques. This field is fundamentally directed toward the analysis of whole genome (genomics), mRNAs (transcriptomics), proteins (proteomics) and metabolites (metabolomics) in biological samples. According to several studies, metabolomics is more effective than other OMICs ّfor various system biology concerns. Metabolomics can elucidate the biological mechanisms of various mesenchymal stem cell function by measuring their metabolites such as their secretome components. Analyzing the metabolic alteration of mesenchymal stem cells can be useful to promote their regenerative medicine application.

Two main properties of stem cells are including prolonged self- renewal and multi-potent differentiation capacity which make them ideal candidate for cell therapy and regenerative medicine (1-5). Related to these properties, stem cells share several essential genes and signaling pathways (i.e. Hedgehog, Wnt, Notch, phosphatidylinositol 3-kinase/ phosphatase, and nuclear factor-κB signaling pathways) as stemness ability (6-8). In other word, stem cells can preserve their lineage, interaction with the environment, and cross-talk with adjacent cells to keep a balance between repose, proliferation, and restoration, through stemness ability (9-11). However, understanding the mechanism of stemness ability is challenging (9). According to several studies, stable, safe, and more accessible stem cells are considered as an excellent choice for regenerative medicine. In this context, mesenchymal stem cells (MSCs) (as easily accessible, self-renewable, and multipotent cells with few consideration ethics) have significant efficacy in regenerative medicine. (12-26). Furthermore, recent development in OMICs approaches (technologies for understanding the whole activity of cells, tissues, and organs at the molecular level) specifically metabolomics approaches (extensive analysis of metabolites in cells, tissues, and organs) can increase our understanding about the self-renewal and differentiation mechanisms. On the other hand, analysis of chemical alterations related to natural processes of living cells including growth, environmental adaptation, and differentiation can be provided by metabolomics methods (27-29).

## OMICs - based stem cell monitoring

**Fig. 1:**
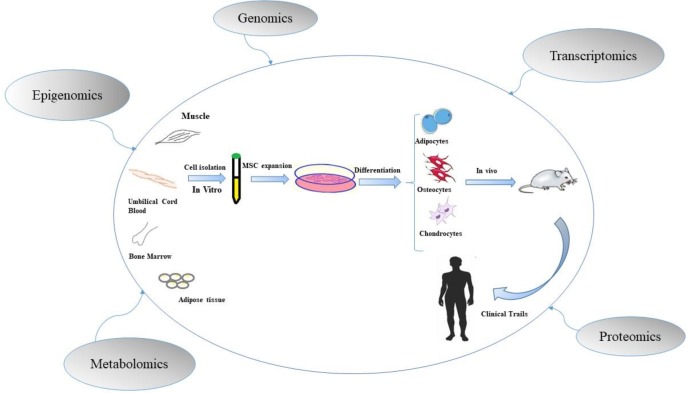
Based stem cell monitoring. Multi- OMICs approaches are functional methods to study stem cell biology and its therapeutic application through evaluation of molecular mechanisms of stem cells properties and quantification of cellular products (33).

Multi- OMICs approaches including geno-mics, epigenomics, transcriptomics, proteomics, and metabolomics are functional methods to study stem cell biology and its therapeutic application (Fig.1) (30-32). 

At first, human genome project has led to the advancement of genome sequencing and study on DNA by analysis of single nucleotide polymorphisms (SNPs), variation copies, and mutations (34-36). Nowadays, genomics as the most mature approache of OMICs and next generation sequencing (NGS) as the latest technology in this field are used for high-throughput  detection and cost effective analysis of biological data (37-40). On the other hand, epigenetic modifications (e.g. methylation and histone acetylation) have an important role in differentiation and development of stem cells (41, 42). The study of heritable modifications (not sequence changes) of DNA is called epigenomics (43, 44). Additionally, qualitative and quantitative transcriptomics can facilitate the investigation of RNAs in stem cells, via molecular and cellular methods such as micro-array and RNA-sequencing (45, 46). It also has a vital role in analyzing key genes and pathways that participate in self-renewal, proliferation, and differentiation of stem cells (47-49). Some transcription factors (related to non-coding RNAs) such as octamer-binding transcription factor 4 (OCT 4) and NANOG can regulate pluripotency feature of stem cells (50, 51). Proteomics tries to evaluate the qualitative and quantitative changes in proteins and identify new markers in stem cell development stages (52, 53). Finally, metabolomics measures and demonstrates the products of metabolism such as amino-acids and fatty-acids. In this respect, metabolomics is an accurate approach to recognize metabolite biomarkers in biological samples (54, 55). Although, application of OMICs, especially metabolomics, for monitoring of stem cell in researches and therapies is in its infancy period, it can be useful to understand different features of cell-based therapy (1, 56).

## Stem cells metabolomics

Because of the self-renewal and differentiation properties of stem cells, they can be applied for regenerative medicine, drug screening, toxicity testing, and evaluation of disease phenotypes (57-59). Although they are metabolically inactive population in quiescent state, their metabolic activity increases during differentiation (60). Stem cells niche can preserve them in a quiescent state to maintain their self-renewal ability (61, 62). In other words, morphogens and growth factors in the niche of stem cells can change the regulation of stem cells through numerous metabolic pathways (1, 63, 64). Moreover, molecular mechanisms can regulate differentiation and reprogramming, and also they can control the energy of metabolism in stem cells throughout glycolytic or oxidative phosphorylation (OXPHOS) reactions (1, 65, 66). In other words, changes in glycolysis and OXPHOS have impact on differentiation or reprogramming of stem cells (66-68). Glycolysis and OXPHOS changes can alter the metabolite levels and reduction–oxidation (redox) state (69-71). Subsequently, hypoxia, glycolysis and redox states can affect the homeostasis and regeneration of stem cells (67, 72, 73). For instance, hypoxia has a key role in maintaining undifferentiated state of stem cells by reducing redox state (74-76). For preparing a balance between self-renewal and differentiation ability, the role of redox state can be important (77, 78). Moreover, the increase of reactive oxygen species (ROS) can promote cell differentiation (74, 79). Herein, understanding the mechanism of stem cells (e.g. MSCs) function is momentous for *in vitro* and *in vivo* studies and also the stem cells application in cell therapy.

## Metabolomics- based comparison of mesenchy-mal stem cells

MSCs as multi-potent stem cells can be extracted from different sources. Their intrinsic properties have drawn the attention for developing more comprehensive studies (13, 14). Moreover, realizing the biological mechanisms of their function can be helpful for developing stem cell researches. Accordingly, metabolomics as a valuable tool for stem cell monitoring can clarify the biological mechanisms of MSCs function through assaying metabolites. Metabolites of MSCs are involved in metabolic or signaling pathways (80-82). Metabolic pathways produce vital signals for the self-renewal, differentiation and other properties of MSCs. On the other hand, undifferentiated state and differentiated state of MSCs can be distinguished via their metabolic profile. Accordingly, in undifferentiated state, mitochondrial OXPHOS is maintained at a low level, while the glycolytic function is maintained at a high level (81, 83). Additionally, in the early phase of MSCs differentiation, down-regulation of some pluripotent genes, up-regulation of terminal genes, and changing the subsets of metabolic enzymes can redirect the new fate of cells. Furthermore, in normoxic states, the proliferation and colony-forming abilities of MSCs are considerably increased (84, 85). In other words, hypoxic condition restricts MSC proliferation to maintain long-term self-renewal capacity. Generally, metabolomics can analyze the rapid kinetics and dynamics of metabolic reactions in different MSCs (86-88). Different types of MSCs share various properties due to their gene expression profile. Additionally, MSCs from various sources have also various secretome and metabolic profile (89, 90).

## Metabolomics analysis of mesenchymal stem cells secretome

MSCs have demonstrated a pivotal and therapeutic impact on several diseases by producing a broad spectrum of autocrine and paracrine secretion factors (secretome) (15, 81, 91). The characterization of the MSCs secretome can elucidate their activation mechanism (92). Accordingly, metabolomics analyses can decipher the mechanism of secretome component functions (93). MSCs conditioned media (MSCs-CM) and extracellular vesicles (EVs) are two main MSC-sourced secretome.

## Metabolomics study of mesenchymal stem cells

**conditioned media**

MSCs-CM encompasses multiple growth factors (GFs), metabolites, and cytokines. It can be prepared through 4 steps including isolation and characterization of cells, culture of cells in a proper culture medium, cell expansion, and CM collection (94, 95). Additionally, it has been shown that MSCs-CM can improve various pathophysiology hallmarks of diseases e.g. lung injury, skin wound, Alzheimer’s disease, and Parkinson’s disease. For instance, there are some anti-inflammatory cytokines in MSC-CM (i.e. ciliary neurotrophic factor (CNTF), transforming growth factor 1 (TGF1), neurotrophin 3 (NT-3) factor, interleukin (IL) 13, IL18 binding protein (IL18BP), IL10, IL17E, IL27 or IL1 receptor antagonist (IL1RA)), and also some pro-inflammatory cytokines (including IL1b, IL6, IL8, and IL9) (95, 96). The equilibrium between these two types of cytokines can mediate the anti-inflammatory impact of MSC-CM. On the other hand, MSC-CM has anti-apoptotic activity via reducing the pro-apoptotic factors and increasing the expression of pro-angiogenic factors. Metabolomics can support quantification of MSC-CM metabolites by different targeted and non-targeted methods (91).

## Metabolomics profiling of mesenchymal stem cells derived extracellular vesicles

EVs including exosomes and micro -vesicles can be secreted by cells which have an important role in intercellular signaling pathways (15, 97). It has been confirmed that MSC-EVs specifically MSCs-derived exosomes (MSC-Exo) can imitate their origin MSCs therapeutic effects in improvement of different disorders. MSC-EVs carry lipids, genetic materials (mRNA and non-coding RNA), and proteins. Moreover, they can be characterized by some surface markers such as CD29, CD73, CD44, and CD105. On the other hand, it is remarkable that MSCs- EVs from different MSC sources have also different composition (98). Namely, menstrual fluid derived MSCs -Exo has greater neurite outgrowth response than bone marrow (BM), chorion, and umbilical cord-derived MSCs. Metabolomics techniques can be used to analyze the mechanism of different MSC-EVs activity based on their different metabolic profile (99).

## Analytical techniques in metabolomics analysis

Metabolomics can assay the metabolite compositions of cells and biological fluids through various targeted and non- targeted techniques (100, 101). A broad range of analytical methods containing capillary electrophoresis (CE) (the separation method in which metabolites are separated based on their migration in the electrical field of the capillary tube), gas chromatography (GC) (a method for separating volatile matters), ultra-performance liquid chromatography (UPLC) (as a modern liquid chromatography method can be used for particles less than 2 µl in diameter), and high performance or high-pressure liquid chromatography (HPLC) (the highly advanced form of column chromatography which pumps the sample of metabolites in mobile phase at high 

**Table 1:** Advantages and disadvantages of metabolomics techniques

**Method**	**Advantages**	**Disadvantages**	**References**
**NMR**	- Simple sample preparation -Excellent reproducibility -Quantify a wide-range of organic compounds in the micro-molar range	-Low sensitivity compared with MS methods - Suitable for quantification of metabolites present in relatively high concentration	(102, 103)
**GC-MS**	- High separation efﬁciency - The oldest and a robust tool for qualitative metabolic profiling	-Non-volatile matrices require additional preparation - Some gases are challenging (CO2, N2, O2, Ar, CO, H2O)	(104, 105)
**LC-MS**	- High separation efﬁciency - No derivatization is needed for the analysis of polar or high molecular weight metabolites - Quick analysis of small samples	- Ion suppression	(103, 106)
**CE-MS**	-Suitable for the separation of polar and charged compounds - Powerful for charged metabolites -High-analyte resolution – providing information mainly on polar or ionic compounds -Short analysis time -Very small sample requirement	- Poor concentration sensitivity	(107, 108)
**HPLC-MS**	-Robustness -Ease of use - Good selectivity -Adjustable sensitivity	-Lack of efficiency due to low diffusion coefficients in liquid phase	(109, 110)
**UPLC-MS**	-Powerful technique in biomolecular research - Covers a number of polar metabolites and enlarges the number of detected analytes -Better efficiency with speedy analysis	Less time life of columns	(107, 111)


pressure within a column or the stationary phase) linked to high-throughput techniques including nuclear magnetic resonance (NMR) (a spectroscopic procedure to follow local strong stationary magnetic fields around atomic nuclei which is for absorbing very high-frequency radio waves) and mass spectrometry (MS) (an analytical manner to ionizing chemical samples to identity unknown composites and chemical features of different molecules based on their mass-to-charge ratio) can be used for separation, examination, and quantiﬁcation of the cellular metabolites composition as metabolomics approaches (107, 112-114). Each of the metabolo-mics approaches has some advantages and disadvantages (Table 1).

## Conclusion and future perspectives

Metabolomics is an impressive research area, which can be used for screening the metabolic modifications during the stem cells reprogramming, proliferation, and differentiation (56, 115). Indeed, screening the metabolic modifications of stem cells (e.g. MSCs) can facilitate their application for regenerative medicine purposes via increasing the man control over *in vitro* manipulation of stem cells including tissue-specific stem cells activation, and promote stem cells for migration to the side of tissue injury. Based on researches, some important metabolic elements can be used to dedifferentiate stem cells toward organ-specific somatic cells (116). Accordingly, in the coming future it seems that the application of generated knowledge on metabolic key methods can be useful for therapeutic targets without the necessity of genetic manipulation. On the other hand, combination of metabolomics technology with other technologies (i.e. genomics, proteomics, structural biology and imaging) can increase its performance to identify novel biological pathways in mechanism of stem cell function, and also to identify disease mechanism (39, 117). Additionally, progress in the development of metabolite databases and *in silico* fragmentation tools can pave the way for large-scale metabolomics analysis (118, 119).
